# A new approach for the treatment of subthreshold bipolar disorders: Targeted high dose levothyroxine and repetitive transcranial magnetic stimulation for mitochondrial treatment

**DOI:** 10.3389/fpsyt.2022.976544

**Published:** 2022-10-13

**Authors:** Andy Zamar, Abbi Lulsegged, Christos Kouimtsidis

**Affiliations:** ^1^Consultant Psychiatrist, The London Psychiatry Centre, London, United Kingdom; ^2^Consultant Endocrinologist Health 121 Ltd., London, United Kingdom; ^3^Honorary Senior Lecturer Imperial College London, London, United Kingdom

**Keywords:** subthreshold bipolar disorder, treatment, levothyroxine, rTMS, mitochondria

## Abstract

Bipolar spectrum disorder includes Bipolar I, Bipolar II and subthreshold bipolar disorders (BD). The condition is highly prevalent, disabling and associated with high mortality. Failure of diagnosis is high. Subthreshold presentations present as 4 or more changes in polarity, are generally less responsive to standard treatment and as a result, drug combinations are often needed. High Dose Levothyroxine (HDT) has been reported to be safe and effective with this condition. Treatment response has been associated with mutations in thyroid activating enzymes and intra cerebral transporter protein carrier. Repetitive Transcranial Magnetic Stimulation (rTMS) has been shown to be effective in bipolar depression and has been proved to have neuroplastic effect. Present authors had reported clinical evidence of safe and effective use of a combination treatment protocol. Potential mechanisms of action of the combined treatment protocol and the role of mitochondria function are discussed.

## Introduction

Bipolar Disorder (BD) is a common (1) and disabling mental illness with significant morbidity and mortality (2). The lifetime prevalence of suicidal risk for people with BD is 15 higher than rest of population and 68.3% of people experience severe impairment in work, family and social function (3). Mortality rates are very high, mainly through Cardiovascular disease (CVD) with 38% CVD deaths 10–20 years before the general population, and 18% through suicide or accidents (4). BD presents in different types, namely Bipolar Disorder I (BD I), Bipolar Disorder II (BD II) and Bipolar Disorder Non-Otherwise Specified (BD NOS) (or subthreshold BD) with different prevalence in the population (BD I and BD II of 1% each and BD NOS of 2.4%), making BD NOS/Subthreshold type the most common presentation (1). Subthreshold or unspecified BD (ICD-10 F31.9) is diagnosable when sufferers exhibit major depressive episodes with manic symptoms but failing to meet the criteria for BP I and II (1). Bipolar I and II also present with subthreshold presentations which are resistant to quetiapine combined with mood stabilizers (5), posing a real challenge to patients and clinicians alike.

The understanding of BD is evolving over the years with changes into the definition and criteria for classification. DSM 5 refers to Bipolar and Related disorders and describes BD I, II, NOS and Cyclothymic disorder (6), and includes a discreet category of Substance/medication induced, Other Specified and Unspecified Bipolar and Related Disorders as well as disorder due to another medical condition (6). There is controversy if rapid cycling (4 or more mood episodes or changes of polarity within 12 months) is a discrete category or a temporal presentation within other categories, with factors such as use of antidepressant medication, use of alcohol or stimulants precipitating presentation (7). DSM 5 acknowledges rapid cycling as presentation of BD I and II (6), although arguably, subthreshold presentation, would be rapid cycling with 4 or more changes in polarity, with short lived phases of hypomanic, mixed and depressive symptoms. Therefore, the presence of short-lived manic symptoms in subthreshold BD can be viewed as changes of polarity, hence are rapid cycling (8). The diagnoses of BD can be complicated by factors such as substance use comorbid disorder and could be difficult to be differentiated from schizoaffective disorder, major depressive disorder, or personality disorder (6). To that effect the term Bipolar Spectrum Disorders (BSD) has been proposed to include all forms of disorder in a continuum that helps both research and clinical management (9).

In this paper we discuss the challenges and gaps in existing guidelines for the treatment of BSD, focusing on subthreshold presentations, the evidence supporting the use and the hypothesized mechanisms of action of Repetitive Transcranial Magnetic Stimulation (rTMS) and in more extent High Dose Levothyroxine (HDT). Finally, we present a new treatment protocol for the combined use of rTMS and HDT and the hypothesized mechanism of action namely restoration of mitochondrial dysfunction.

## Challenges and limitations of existing treatment options

Alongside the evolvement of understanding of the BSD, treatment guidelines are evolving too. Whereas there is an overall agreement across guidelines regarding the treatment of BD I, BD II, there is far less scientific evidence and certainty regarding the treatment of the rest of the categories (subthreshold presentations) (10).

For example, there is consensus for the role of Lithium as a long-term prophylactic treatment option for BD I, the importance of withdrawal of antidepressants and elimination of lifestyle factors that might contribute to the course or have an impact on pharmacological treatment for both I and II (11). In general, most people would require short term or long-term combination of medications, with an average of 3.8 medications (12). Treatment-emergent affective switches are an issue when managing bipolar depression (13). Discontinuation rates with lithium and lamotrigine are reported to be very high even up to 76% (14) and caution is suggested for use of Valproate with women during reproduction age (15). Weight gain reported to be the main side effect associated with the use of antipsychotic medication and tachyarrhythmias, though very common with almost 1 in 4 seem to be under reported (16). Numbers Needed to Treat (NNT) for response is between 5 and 12 in antipsychotics (17), although full remission should be the standard outcome in BD and not just response (18).

The National Institute for Health and Care Excellence (NICE) guidelines make recommendations for the treatment for mania (BD I) or hypomania (BD II), treatment for bipolar depression and maintenance treatment (19). NICE specifically excluded Unspecified (subthreshold) BD, despite it being the commonest and equally disabling presentation. For Rapid Cycling presentations, they recommend offering the same interventions as for other types of BD “because there is currently no strong evidence to suggest that people with rapid cycling BD should be treated differently” (19). There is, however, evidence showing that rapid cycling and subthreshold symptoms should not be treated as non-rapid cycling BD (5, 12, 13). For bipolar depression they make three recommendations for pharmacological treatment (a) Olanzapine and Fluoxetine combination, (b) Quetiapine and (c) Lamotrigine (19), the latter may be of dubious efficacy (15). Maintenance treatment is recommended to reduce the frequency and severity of relapses as well as to protect for worsening of cognitive function and overall functionality (19).

The American Psychiatric Association (APA) STEP-BD program proposed a three steps approach to the treatment of Rapid Cycling presentations with (i) identification and minimization or elimination of pro-cycling factors, (ii) then optimization and if necessary, add of mood stabilizers and/or second-generation antipsychotics, and (iii) invoke novel treatments such as Levothyroxine or ECT (20). The Maudsley Prescribing Guidelines in 2018 advise clinicians to consider adjunctive rTMS but indicate no preference for one strategy (21). This recommendation does not appear in the most recent publication of 2021 (8). Canadian Network for Mood and Anxiety Treatments (CANMAT) and International Society for Bipolar Disorders (ISBD) 2018 guidelines recommend rTMS as third-line treatment in acute mania (right side) and acute bipolar I depression (left or right), and non-specific recommendation for BD II depression (11). Thyroid hormones are also recommended as third-line treatment for acute BD I depression (11) but at the time there was no positive randomized controlled trial (RCT). Electro Convulsive Treatment (ECT) is recommended for management of acute mania, acute depression in BD I and II (11) and for the treatment of mania when resistant to any other treatment (8, 11, 19).

In conclusion the understanding of diagnosis and different forms of BD has evolved over the years. It is reported that subthreshold presentations are far more common than the typical type I and II combined. We argue that there is a paradox concerning the existing treatment evidence for the disorder. The amount of evidence of existing treatment options of what is described as subthreshold presentations, including rapid cycling presentations, is limited. Existing guidelines seems not to pay appropriate attention to this knowledge gap. Existing evidence reports poor compliance with proposed treatment options due to high incidence of side effects, also possible due to the combination of several medications and medication induced mood switch. Furthermore, it is well documented that rate of relapse is high. Hence the urgent need for better understanding of the condition and the need for treatment innovation.

## The evidence for repetitive transcranial magnetic stimulation

Repetitive transcranial magnetic stimulation (rTMS) is a non-invasive, brain stimulation therapy with growing evidence for its use in bipolar depression. A right-side DLPFC protocol was better than left side with NNT for response of 3 and response in 60 vs. 6.6% in sham (228). Low rates of treatment emergent affective switches were reported (22). A network analysis (113 trials/6,750 patients) of major depression and Bipolar Depression on non-surgical brain stimulation (ECT, rTMS, Transcranial direct current stimulation (tDCS), and Magnetic Seizure Therapy), provided evidence for the acceptability and the effectiveness of non-surgical brain stimulation techniques as alternative or add-on treatments for adults (23), as well as the need for further well-designed research (23, 24). Systematic review and meta-analysis of rTMS for Bipolar Depression indicated that it is safe and effective with low-risk side effects (24, 25). Furthermore, depressed patients with a diagnosis of BD benefit from rTMS in a similar fashion as patients with unipolar depression in a naturalistic setting (26).

## The mechanism of action of repetitive transcranial magnetic stimulation

There is accumulating evidence to suggest that the effect of rTMS on brain leading to the observed clinical benefit is due to a significant neuroplastic effect (27, 28). Neuroplasticity refers to production of neurons, growth of axons and dendrites and the formation and reorganization of synapses (29). These effects could be measured by changes in hippocampal and amygdala volume as well as cortical thickness (30). Despite ongoing uncertainties, evidence from human studies and animal models suggest that the effect is mediated by “long-term potentiation (LTP)-like” mechanisms (27). It is hypothesized that magnetic stimulation with simultaneous (and repeated) activation of pre- and postsynaptic structures, increases excitatory synaptic strength of cortical neurons (27). The role of mitochondria in neuroplasticity has been investigated and it is considered to play important role in controlling fundamental processes including neural differentiation. Mitochondria are highly mobile and move within and between subcellular compartments involved in neuroplasticity (synaptic terminals, dendrites, cell body and the axon) (29). Kindling effect is a recognized feature in BD (30). rTMS showed to increase Cerebral Blood Flow and oxygen utilization (31) which persists after the session (32), leading potentially into reversal of kindling effect.

## The evidence for high dose levothyroxine

**FIGURE 1:**
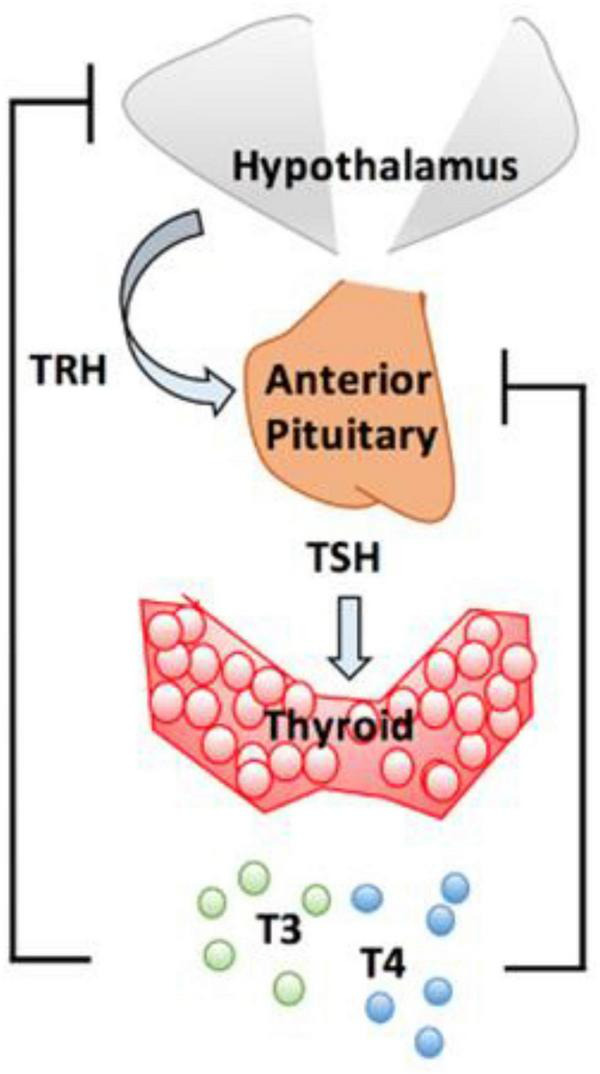
Hypothalamo-pituitary-thyroid axis. The hypothalamus release thyrotrope releasing hormone (TRH) which stimulates pituitary thyrotrope cells to release thyroid stimulating hormone (TSH). TSH promotes production and secretion of T4 and T3. From J. Clin. Med. 2020, 9(6), 1679.

The actions of thyroid hormones are mediated via two principal hormones, thyroxine (T4) and 3,5,3′-triiodothyronine (T3). Secretory control of these hormones is mediated via Thyroid Stimulating Hormone (TSH) secreted by the pituitary gland in response to Thyrotrope releasing hormone from the hypothalamus. T3 is the most active form of thyroid hormone and approximately 20% is produced by the thyroid gland while the majority is derived by peripheral conversion of T4 to T3; Deiodinase type 1 and type 2 (DiO1 and DiO2, respectively), convert T4 to T3 (33). It is through T3 that the effects of thyroid hormone are mediated in target tissues and thus, T4 acts as a pro-hormone. DiO1 is responsible for the majority of circulating T3 while DiO2 is responsible for the provision of intracellular T3. A third deiodinase, DiO3, inactivates T4, converting it into reverse T3 (rT3) and inactivates T3 by converting it into 3,3′-diiodothyronine (T2). Figure 1 shows the Hypothalamo-Pituitary-Thyroid axis.

There are two placebo controlled RCTs using supraphysiological/HDT dosage of thyroxine. A 6-week, double-blind, randomized, placebo-controlled fixed-dose (300 mcg/d) trial, assessed the efficacy and tolerability of levothyroxine as an adjunct to continuing treatment with mood stabilizer and/or antidepressant medication for patients with BD I or II, currently depressed (as per DSM-IV) (34). The primary efficacy variable was mean change in Hamilton Depression Rating Scale (HDRS) score. This trial demonstrated that patients treated with levothyroxine did numerically better than those treated with placebo; however, the study failed to detect a statistically significant difference due to a high placebo response rate (35). A significant shortfall in this study was limiting the dose to 300 mcg, as average doses in the author’s and others are above 300 mcg (36, 37). In an another RCT, 32 treatment-resistant patients with rapid cycling BD who had failed a trial of lithium, were allocated into three treatment arms: levothyroxine, T3, or placebo and were followed for ≥ 4 months with weekly clinical and endocrine assessments (38). The findings suggested a benefit of adjunctive levothyroxine in alleviating resistant depression, reducing time in mixed states and increasing time euthymic. Adjunctive T3 did not show statistically significant evidence of benefit over placebo in reducing the time spent in disturbed mood states (39). There was no dose limit in this study.

Several studies indicated the good tolerability of treatment in BSD (40), as far as comorbid anxiety symptoms (38), lack of any signs of thyrotoxicosis (34, 35, 38–40) even in doses of 600 mcg and 150–1,000 mcg (37, 41). Osteoporosis remains a concern for long-term HDT use despite reassuring data on the contrary (37) with only sporadic cases reported of women with mood disorders having reduced bone mineral density (BMD) on HDT (42).

## Mechanism of action of high dose levothyroxine

### Increasing local production of T3 in the brain

It is unclear how exactly, HDT is of benefit to patients with BSD. A Chinese case-control association study showed that compared to controls, patients with a heterozygote polymorphism of the DiO2 gene (CT allele) had a 1.6-fold increased risk of BD and a 3.75-fold increased risk if they had a homozygous variant (CC allele). Thr92 substitution for Ala substitution in DiO2 (Thr92Ala) is one of the most commonly studied polymorphism of DiO2 (43). In addition to its association with BD, Thr92Ala has been associated with mental retardation, hypertension, type 2 diabetes mellitus and osteoarthritis (43–46).

It is interesting that DiO2 variants will produce a wide range of different phenotypic expressions in individuals. A global DiO2 knock-out mouse model extensively studied for neurological and cognitive effects demonstrated having only reduced agility (47). Yet, with selective inactivation of D2 in the astrocytes (astrocyte-D2 knockout mice), affected mice showed substantial reduction in D2 mRNA, reduced D2 activity (but normal D3 activity) in the cerebral cortex, hippocampus and they exhibited anxiety-depression type behavior with reduced hippocampal expression of markers associated with depression in animal models (48). DiO2 activity was normal in the pituitary and the hypothalamus and circulating thyroid hormone levels were normal. This is similar to the cohort of patients reported by present authors (37)—baseline thyroid hormone levels were normal, yet they had a mood disorder that proved responsive to HDT. Interestingly, the authors comment that the Astrocyte-DiO2-knockout mice model was associated with hippocampal gene expression that modified mitochondrial function and response to oxidative stress and exercise in these mice reduced anxiety and depression (48).

Additionally, animal studies show approximately 80% of intracellular cerebral cortex T3 was derived from local, cellular conversion of T4 to T3 via DiO2 rather than from the circulation (49). Thus, an in adequate supply of the prohormone, T4 could potentially lead to reductions in intracellular concentrations of T3 in specific tissues such as the brain. Further evidence to support this comes from a study in which patients taking levothyroxine monotherapy who also had Thr92Ala variants were shown to improve psychologically when T3 was added in (50). It is therefore postulated that polymorphism of the DiO2 gene could potentially lead to reduced conversion of T4 to T3 resulting in complications such as mood disorders. Present authors previously reported that 95% of 20 consecutive patients with rapid recycling BD had single nucleotide polymorphisms of DiO1, DiO2, or both (37).

### Non-T3 mediated effects

However, *in vitro* studies show that Thr92Ala variants are not associated with reduced activity potentially casting some doubt on the hypothesis that Thr92Ala variants exert their effect by reducing bioavailability of T3 (51). Therefore, another mechanism by which Thr92Ala variants might be phenotypically expressed is via endoplasmic reticulum oxidative stress. Normally, D2 resides in the endoplasmic reticulum and is not found in the trans-golgi apparatus. However, Thr92Ala variants are associated with accumulation of D2 in the golgi apparatus and reduced ability to produce T3 (52).

### Mitochondrial dysfunction

Functional MRI studies have shown that patients with Bipolar disease have markers in keeping with mitochondrial dysfunction and reduced energy metabolism including, decreased pH, phosphocreatine and adenosine triphosphate (ATP) levels and increased lactate (53). Thyroid hormones have been shown to improve mitochondrial biogenesis and production of ATP.

## Explanation of lack of side effects from high dose levothyroxine

It is curious, if not mesmerizing when a clinician encounters a patient taking HDT for BSD for the first time since they are (a) taking very high doses of Levothyroxine, (b) have blood results typically seen in the thyrotoxic range [with certain caveats reported previously (37)] and (c) are essentially clinically, euthyroid. An 8-week study published in 2002 compared the response to HDT in 13 patients with refractory depression with 13 healthy controls (54). The dose of levothyroxine was increased to 500 mcg once daily. The discontinuation rate in the control group was much higher—38% while none of the subjects with refractory depression discontinued treatment. Interestingly, the levels of free T4 and free T3 were higher in control group which could imply a reduced ability to inactivate free thyroid hormones. This small study showed that the physiological environment in patients with bipolar disease is much more receptive to HDT without overt side effects (54). Possible explanations for this picture include the following:

### Robust peripheral inactivation of circulating thyroid hormones

**FIGURE 2:**
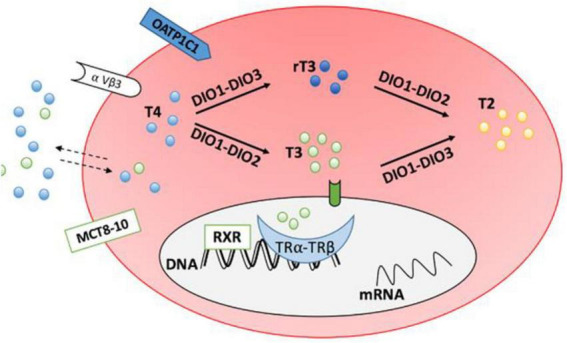
Transporter proteins monocarboxylate transporter 8–10 (MCT8-10) and organic anion transporting polypeptide 1C1 (OATP1C1) facilitate entry of thyroid hormones into cells. DiO1 and DiO2 convert T4 to T3 (DiO2 is responsible for intracellular conversion while DiO1 is extracellular). DiO1 and DiO3 inactivate T4 by converting it to reverse T3 (rT3). From J. Clin. Med. 2020, 9(6), 1679.

The actions of DiO3 (and D1) promptly inactivate HDT in the peripheral tissues leading to increased levels of reverse T3 (rT3). rT3 can further inhibit production of the active hormone, T3 by inhibiting DiO1 and DiO2 (55). Concentrations of reverse T3 were reported by present authors (37). Figure 2 shows the mechanism of inactivation.

Once DiO2 converts T4 to T3, DiO2 is inactivated through a conformational change that is mediated by a ubiquitin protein. Thus, in peripheral tissues, the greater the T4 concentration, the greater the requirement for DiO2 to inactivate it which in turn leads to reduced levels of active DiO2 and ultimately, reduced levels of T3. Thus, the higher the level of T4, the greater the reduction in DiO2 activity which serves to prevent excess, harmful production of T3.

### Reduced tissue levels of T3

The amount of intracellular T3 can vary significantly in different tissues due in part, to the actions of the deiodinase enzymes (33). As an example, Hypothalamo-Pituitary regulation of thyroid hormone synthesis and secretion is interesting; DiO2 is strongly expressed in the pituitary and results in conversion of circulating T4 to T3, and levels of DiO3 are comparatively low (56). Unlike in peripheral tissues, the ubiquitination of DiO2 is less pronounced and thus, there is a greater tendency for DiO2 to remain “active.” T3 production in the hypothalamo-pituitary glands is therefore relatively preserved compared to the peripheral circulation. The Hypothalamus and Pituitary essentially tolerate a higher level of T4 to maintain T3 levels. The situation can, however, be very different if the thyroid gland is dysfunctional or absent.

Evidence from animal and human studies show that different tissues in the body do not respond to levothyroxine in the same manner. A study published in 2011 looking at 1,811 athyreotic patients with normal TSH levels taking levothyroxine monotherapy and 3,875 euthyroid controls showed more than 20% of the group taking Levothyroxine had significantly higher free T4 and lower free T3 levels compared to euthyroid controls, despite having normal TSH levels (57). This suggests that levothyroxine monotherapy might be sufficient to normalize TSH levels (because of adequate central conversion of T4 to T3 in the pituitary and reduced ubiquitination of DiO2) but insufficient to restore normal peripheral circulating free T4 and free T3 levels. A meta-analysis explored this further by incorporating data from 99 studies and showed that Levothyroxine monotherapy, at doses sufficient to normalize TSH in hypothyroid patient, was insufficient at restoring tissue biomarkers of thyroid function such as cholesterol levels, resting energy expenditure, creatine kinase and/or ferritin, sex hormone binding globulin and cognition (58). Thus, it is evident that some patients exhibit different (tissue) responses to levothyroxine.

## Combined high dose levothyroxine and repetitive transcranial magnetic stimulation

The combination of HDT and rTMS is novel, and as the use of rTMS was recommended in the Maudsley prescribing guidelines since 2010, and HDT since 2004, the authors combined both interventions relying specifically on the statement that there is no first-choice drug or combination recommended, in the guideline. The present authors reported data on the combination of HDT with rTMS from 20 patients with rapid cycling BD (therefore BSD) who were on multiple medications at the start of treatment, and they were treatment resistant (37). During the acute phase of treatment eight patients (40%) needed only levothyroxine, 10 patients (50%) needed one additional medication and 2 (10%) needed two additional medications. During the maintenance phase, 11 patients (55%) were maintained only on Levothyroxine. Eight patients (40%) experienced relapse with an average time to relapse of 31.5 months (range 16–65 months). Relapse was triggered by non-compliance with lifestyle adjustments in 3 patients (alcohol and/or caffeine use), PTSD following traumatic events in 3 patients, exams stress in 1 patient and no trigger in 1 patient. Remission in non-relapsed patients was maintained for an average of 57 months (range of 26–145 months) (37).

In a more recent evaluation of 55 patients, from same authors, no patient had to discontinue treatment. Fifty-three patients were in remission (96.4%), with an average duration of 2.0 years (median: 1.5; range: 0.2–6). The mean duration of time to reach initial remission was 42.6 weeks and the average dose of levothyroxine was 303.7 mcg (median: 300; range: 50–600). The average number of medications prescribed including levothyroxine was 2.0 (median: 2, range:1–5). The Sheehan Disability Scale score strongly improved from 7.33 (median: 8; range: 1.4–10) at the start of treatment to 1.27 (median: 0.3; range: 0–8.6) at the time of review (mean change 6.06 (95% C.I. 5.36–6.77), t (54) = 17.23, *p* < 0.001, Cohen’s *d* = 2.61 (95% C.I. 1.81–2.83). Eighteen patients (32.7%) scored 0 at review (personal communication available at request).

## Mechanism of action of combination protocol

The physiological mechanism associated with the reported effectiveness of the combination of HDT and rTMS is not known. It might be associated though with intracellular and intercellular changes associated with the combination of the effect of each of the treatment components.

**FIGURE 3:**
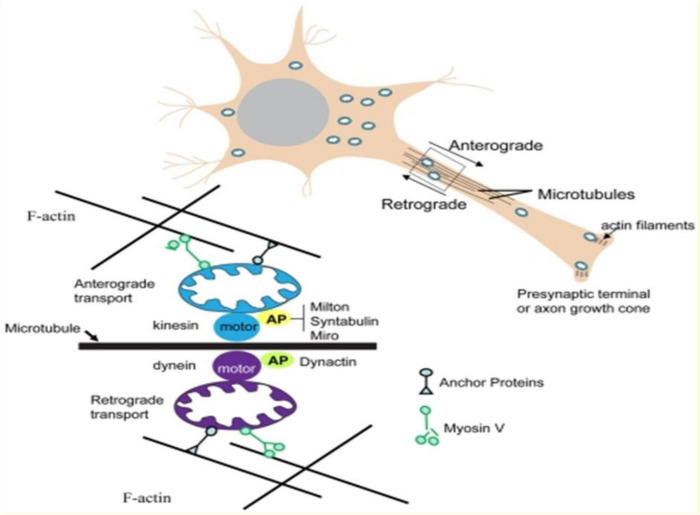
Molecular machinery that actively moves mitochondria to and from within axons.

Mitochondrial dysfunction appears to be central in the pathogenesis of mood disorders and in the treatment of mood disorders. Gimenez-Palomo extensively reviewed the evidence for this connection, and we hypothesize that the mitochondrial dysfunction, at least largely found in BPS disorders may be due to innate intracerebral thyroid deficiency in the brain tissues. They have also highlighted markers of impaired mitochondrial function such as reduced ATP and increased lactate, also in patients with BDs (59). Thyroid hormones stimulate mitochondriogenesis, augmenting cellular oxidative capacity. They also affect the inner mitochondrial membrane, ATP synthesis and turnover reactions (59–63). Alcohol is a well-established mitochondrial toxin (64), whilst stress, is also acknowledged to have a significant effect on mitochondrial function (65), hence the importance of discontinuing alcohol and managing stress in BPS. It could be argued that genetic polymorphism might cause mitochondrial dysfunction at intracellular level, also affected by factors such as alcohol consumption, which is corrected by lifestyle changes and HDT and further enhanced by the optimization of intercellular communication and brain function with the use of rTMS (see Figure 3).

A major mechanism by which mitochondria are transported in either anterograde or retrograde directions in axons involves their energy (ATP)-dependent movement along microtubules. ATP-dependent “motor” proteins interact with the microtubules to generate the force that moves the mitochondria in anterograde (kinesin) or retrograde (dynein) directions, respectively. Several APs (adaptor proteins) mediate the interaction of mitochondria with motor proteins, including APs that interact with kinesin (Milton, syntabulin and the Rho GTPase Miro) and APs that associate with dynein (dynactin). In addition, in synaptic terminals and growth cones, microtubules may be moved by myosin-mediated interactions with actin filaments. Myosin V can drive short-range movements along F-actin, as well as modulate long-range transport by pulling mitochondria away from microtubules by facilitating anchorage of mitochondria to F-actin by unknown actin–mitochondrion crosslinkers [adapted from Mattson et al. (66)].

In simpler terms, in the combination protocol of HDT/rTMS, HDT stimulates mitochondrial generation and activity whilst rTMS induces neuroplasticity. Mitochondria play an integral role in neuroplasticity, and together they enhance and sustain remission thus countering the kindling effect in mood disorders.

## Conclusion

The authors argue that there is urgent need to focus scientific attention on the understanding of physiological mechanisms associated both with the etiology of BD as well as the effect of innovative treatment approaches. A combination of HDT and rTMS seems to be a promising treatment innovation, based on valid preclinical evidence of both genetic factors, physiological mechanism underlying the condition and explaining treatment effect. Present authors argue that the focus of treatment should be on neuronal mitochondria, rather than neurotransmitters, and have reported on efficacy, safety and compliance due to low incidence of side effects and reduced risk of relapses across the bipolar spectrum with rapid cycling, a state denoting poor response and prognosis.

## Author contributions

AZ developed the treatment protocol and contributed to the literature review and writing up of the manuscript. AL contributed to the literature review. CK contributed to the literature review and had main responsibility for writing the manuscript. All authors contributed to the article and approved the submitted version.
